# Linked administrative health data on prehospital olanzapine administration by paramedics in Winnipeg, Canada: Challenges and opportunities

**DOI:** 10.23889/ijpds.v9i5.2802

**Published:** 2024-09-18

**Authors:** Gilles Detillieux, Neil McDonald, Jennifer Enns, Chelsey McDougall, Nathan Nickel

**Affiliations:** 1 Manitoba Centre for Health Policy, University of Manitoba; 2 Winnipeg Fire Paramedic Service, City of Winnipeg

## Objective

Olanzapine is an antipsychotic drug used in emergency departments to treat methamphetamine intoxication. Our study aim is to examine whether prehospital olanzapine administration by paramedics in Winnipeg, Canada, improves outcomes for individuals experiencing methamphetamine intoxication. However, the ‘real-world’ nature of the administrative data has presented several challenges.

## Approach

First, we needed to determine whether individuals experiencing methamphetamine intoxication received or did not receive olanzapine. The paramedic records identify whether olanzapine was administered but not whether individuals were considered for olanzapine without receiving it. To address this, we manually reviewed the unstructured narratives from the paramedic assessments. 8000+ records were independently evaluated by two experienced paramedics to determine eligibility. Disagreements were resolved by a paramedic-educator.

Second, we discovered that ~40% of paramedic records for 2019 did not link correctly to other health data. We conducted a sensitivity analysis to assess the impact of excluding records with incorrect linkage, but the loss of records in the most critical year of assessment led us to reject this approach. Instead, we revisited the original data linkage to identify and correct the cause of the errors.

## Results

We have constructed a cohort that allows us to compare treated (n=222) and untreated (n=205) individuals experiencing methamphetamine intoxication and provides enough statistical power to assess the impact of prehospital olanzapine treatment on hospital outcomes.

## Conclusion & Implications

Administrative data from the real world are powerful tools for research with potential to show important health impacts, but their use requires creative thinking to overcome unexpected data challenges.

